# Enhancing long-term forecasting: Learning from COVID-19 models

**DOI:** 10.1371/journal.pcbi.1010100

**Published:** 2022-05-19

**Authors:** Hazhir Rahmandad, Ran Xu, Navid Ghaffarzadegan

**Affiliations:** 1 Sloan School of Management, Massachusetts Institute of Technology, Cambridge, Massachusetts, United States of America; 2 Department of Allied Health Sciences, University of Connecticut, Storrs, Connecticut, United States of America; 3 Department of Industrial and Systems Engineering, Virginia Tech, Falls Church, Virginia, United States of America; Fundação Getúlio Vargas: Fundacao Getulio Vargas, BRAZIL

## Abstract

While much effort has gone into building predictive models of the COVID-19 pandemic, some have argued that early exponential growth combined with the stochastic nature of epidemics make the long-term prediction of contagion trajectories impossible. We conduct two complementary studies to assess model features supporting better long-term predictions. First, we leverage the diverse models contributing to the CDC repository of COVID-19 USA death projections to identify factors associated with prediction accuracy across different projection horizons. We find that better long-term predictions correlate with: (1) capturing the physics of transmission (instead of using black-box models); (2) projecting human behavioral reactions to an evolving pandemic; and (3) resetting state variables to account for randomness not captured in the model before starting projection. Second, we introduce a very simple model, SEIRb, that incorporates these features, and few other nuances, offers informative predictions for as far as 20-weeks ahead, with accuracy comparable with the best models in the CDC set. Key to the long-term predictive power of multi-wave COVID-19 trajectories is capturing behavioral responses endogenously: balancing feedbacks where the perceived risk of death continuously changes transmission rates through the adoption and relaxation of various Non-Pharmaceutical Interventions (NPIs).

## Author summary

Long-term projections of COVID-19 trajectory have been used to inform various policies and decisions such as planning intensive care capacity, selecting clinical trial locations, and deciding on economic policy packages. However, these types of long-term forecasts are challenging as epidemics are complex: they include reinforcing contagion mechanisms that create exponential growth, are moderated by randomness in environmental and social determinants of transmission, and are subject to endogenous human responses to evolving risk perceptions. In this study we take a step towards systematically examining the modeling choices that regulate COVID-19 forecasting accuracy in two complementary studies. First, we leverage the diverse models contributing to the CDC repository of COVID-19 USA death projections to identify factors associated with prediction accuracy across different projection horizons. Second, we design a very simple forecasting model that only incorporates the key features identified in the first study, and show that the long-term prediction accuracy of this model is comparable with the best models in the CDC set. We conclude that forecasting models responding to future epidemics would benefit from starting small: first incorporating key mechanistic features, important behavioral feedbacks, and simple state-resetting approaches and then expanding to capture other features. Our study shows that the key to the long-term predictive power of epidemic models is an endogenous representation of human behavior in interaction with the evolving epidemic.

## 1. Introduction

From public policy to strategic management, forecasting long-term trajectories is inevitable: planning for the future rests on forecasts [1,2] and models, mental or formal, must be used to produce those forecasts. Yet long-term forecasts are difficult, especially when systems’ dynamics are regulated by human action [3,4]. What features of predictive models for such systems enhance their long-term accuracy? The COVID-19 pandemic offers a natural testbed to address this broad question in the context of a high-stake problem. Long-term projections of pandemic trajectory have been used to inform planning intensive care capacity, selecting clinical trial locations, deciding on economic policy packages, and many other consequential decisions, as well as individual choices on careers, vacations, home purchases, investments, and others. Prediction is not the primary purpose for many models [5], but a large number of simulation and machine learning models have been developed to predict the trajectory of the disease at different geographic resolutions in order to inform individual and policy responses [6–11]. These models have offered significantly different predictions over time: some early models predicted millions of deaths during the first few months in the United States [7], while others expected an end to the pandemic by May 2020 [8,12]. While predictions began to become more comparable with the increasing knowledge about the disease, policy makers seeking guidance on tough, urgent, questions would nevertheless get divergent answers from the existing models, leading to criticism of the established modeling approaches [13–15]. In fact, some studies suggested that attempts at predicting epidemic trajectories over the longer term will inevitably fail due to the combination of exponential growth and stochastic shocks inherent to the dynamics of contagion [16,17]. Moreover, the complexity of predicting what policies will be adopted in the future by various communities have remained as a major challenge to longer-term epidemic projections. In response, and with a few exceptions [8,9,18], most later models have focused on predictions that span only a few weeks, leaving out the long-term multi-wave dynamics observed across nations and central to many policy and personal questions. Even researchers actively leading COVID-19 projection efforts increasingly emphasize the many challenges involved [19].

Existing predictive models of COVID-19 vary significantly in their methodological approach, level of complexity, the mechanisms explicitly modeled, how human behavior is modeled, use of data, and their estimation and projection frameworks. This diversity indicates differing working theories across the research community on what it takes to build a good predictive model. Designing, or even selecting, an appropriate model is made difficult by the lack of agreement about the features of models that are critical for useful predictions. Understanding the relative value of different modeling assumptions and constructs would also inform the more promising areas of research that would enhance future models of epidemics and beyond.

In this paper, we take a step toward systematically examining the modeling choices that regulate forecasting accuracy. The paper includes two complimentary studies. First, we use data from the Centers for Disease Control and Prevention (CDC) repository of COVID-19 projections to understand the drivers of forecast accuracy. We focus on 490,210 point-forecasts for weekly death incidents (across 57 locations); forecast dates over the span of a year (4/13/2020 to 3/29/2021); 20 forecast horizons (1-week-ahead predictions to 20-week-ahead predictions); and 61 models (not every model offers a forecast for every location, forecast date, or horizon). Prior research has used these data to assess the quality of probabilistic forecasts from various models [20,21]. We build on this work by examining the association between the modeling approach and point prediction quality using hand-coded architectural features of forecasting models. Those associations are not causal, but inform a set of hypotheses on the features that *may* enhance the predictive accuracy of models.

Second, we test those hypotheses by designing a simple model that focuses only on capturing the identified features and leaves out many other prominent features. We assess the predictive performance of the resulting model and quantify the marginal value of each proposed feature by comparing projections from an alternative model that excludes the feature. This simple model, referred to as SEIR*b*, enhances the conventional models by explicitly capturing how *behavioral* responses of individuals and societies condition the evolution of a pandemic, and it can be used as a base for developing more accurate models for epidemic forecasting.

## 2. First study: Associations between model architecture and predictive accuracy

### 2.1. Methods

The data for the first study come from the CDC collection of COVID-19 forecasts. In April 2020, and with the growing need to forecast COVID-19 trajectories in the United States, the CDC partnered with a research laboratory at the University of Massachusetts Amherst to create a forecast hub that collects and synthesizes COVID-19 trajectory predictions [20]. As of May 2021, more than 70 modeling groups from academic institutions, research laboratories, and the private sector had contributed by submitting their simulation-based trajectory predictions for various locations in the United States (typically at the state and national levels, but also some county-level predictions). Each week, each individual model’s predictions (and confidence intervals) and an ensemble of the collective results were reported on the CDC’s website. Some of these teams provided only short-term predictions, in the range of 1–4 weeks, while others provided longer projections, mostly up to 8 weeks and a few up to 20 weeks ahead. Some 61 models provide death projections. These models and their weekly death predictions are the primary data we use for the first study.

#### Forecast Data

For the project, we collected the forecast data from the inception of the CDC forecast hub through 3/29/2021 from the project’s GitHub site. We stopped in March 2021 because the appearance of new variants (Delta in May 2021; Omicron in December 2021), along with mass vaccination, have since had major impacts on the trajectory of the pandemic, and a careful analysis of modeling choices on vaccination and variants deserves a separate study. We focus our analysis on the accuracy of death projections because of the higher degree of accuracy of death data (compared to reported infections) and the fact that more of the models predict deaths. We also focus on the accuracy of point estimates rather than prediction intervals because prediction interval accuracy is a distinct and complex outcome that may depend on factors different from those determining point prediction accuracy (e.g. the flexibility of likelihood functions used in estimation). While it is feasible to combine point predictions and prediction intervals using proper scoring rules [22], we maintain our focus distinctly on drivers of point prediction accuracy and omit a comparison of prediction intervals (e.g., see [20,21] for comparing those intervals). We collected all the state- and national-level forecasts we could find on the project website. Therefore, our data structure includes one prediction (of expected number of deaths) for each model (a total of 61 models), location (a total of 57 locations spanning the U.S. states and territories, as well as the nation *in total*), forecast date (4/13/2020 to 3/29/2021), and forecast horizon (1 to 20 weeks ahead). A total of 490,210 such forecasts from the CDC hub inform our comparisons. In study two those data are augmented by analogous forecasts from the model(s) we build in that study. We downloaded the actual death data through 5/17/2021 from the same website, which curates the data from The Johns Hopkins University’s COVID-19 statistics hub [23].

#### Coding Model and Estimation Architectures

We need to identify relevant features of the models studied to associate models’ architecture with their predictive accuracy. However, the models included on the CDC hub span different methodological approaches, use different data sources, have different levels of complexity (in the number of variables, computational costs, and mechanisms they explicitly represent), and vary in their estimation and projection approaches. This heterogeneity complicates the task of specifying standard assessment dimensions applicable to all models; features relevant for some model types, such as whether hospital capacity allocation is explicitly modeled, may be relevant for a mechanistic model but arguably irrelevant for a prediction model using neural networks. Moreover, as one zooms into the modeling details, the number of features that could potentially be associated with predictive performance multiply. Any statistical analysis associating the performance of some 60 models with dozens of features is likely to generate spurious correlations rather than generalizable insights. A more pragmatic challenge in identifying relevant features of the models is the large variation in the quality of documentation for the existing models: while some include extensive documentation and opensource codebases, others may only have a few lines of explanation about the underlying model and estimation architecture.

With these challenges in mind, we focused on identifying high-level architectural features that could be identified for most models on the hub. Specifically, we coded each model into one of the four categories: mechanistic compartmental (with and without state resetting; see explanation below); non-mechanistic; ensemble (of multiple models); and other models (including two individual-level models, only one providing death projections). Other coded features were data inputs, output variables, approach to projecting future trajectory of the reproduction number or policy interventions, and capturing mobility, as well as general information such as the modelers’ affiliation (academic or non-academic), disciplinary background, and the availability of technical documentation. For mechanistic models with adequate documentation, we looked into the details of the compartmental structure and whether they included coupled compartments with commuting across regions or age-related structures. Furthermore, we documented their parameter estimation approaches. For non-mechanistic models with adequate documentation, we separated simple regression models from more sophisticated curve-fitting approaches and machine learning techniques. For ensemble models, we coded the types of models used in the ensemble. S1 Text provides additional details.

### 2.2. Results

**Fig 1:**
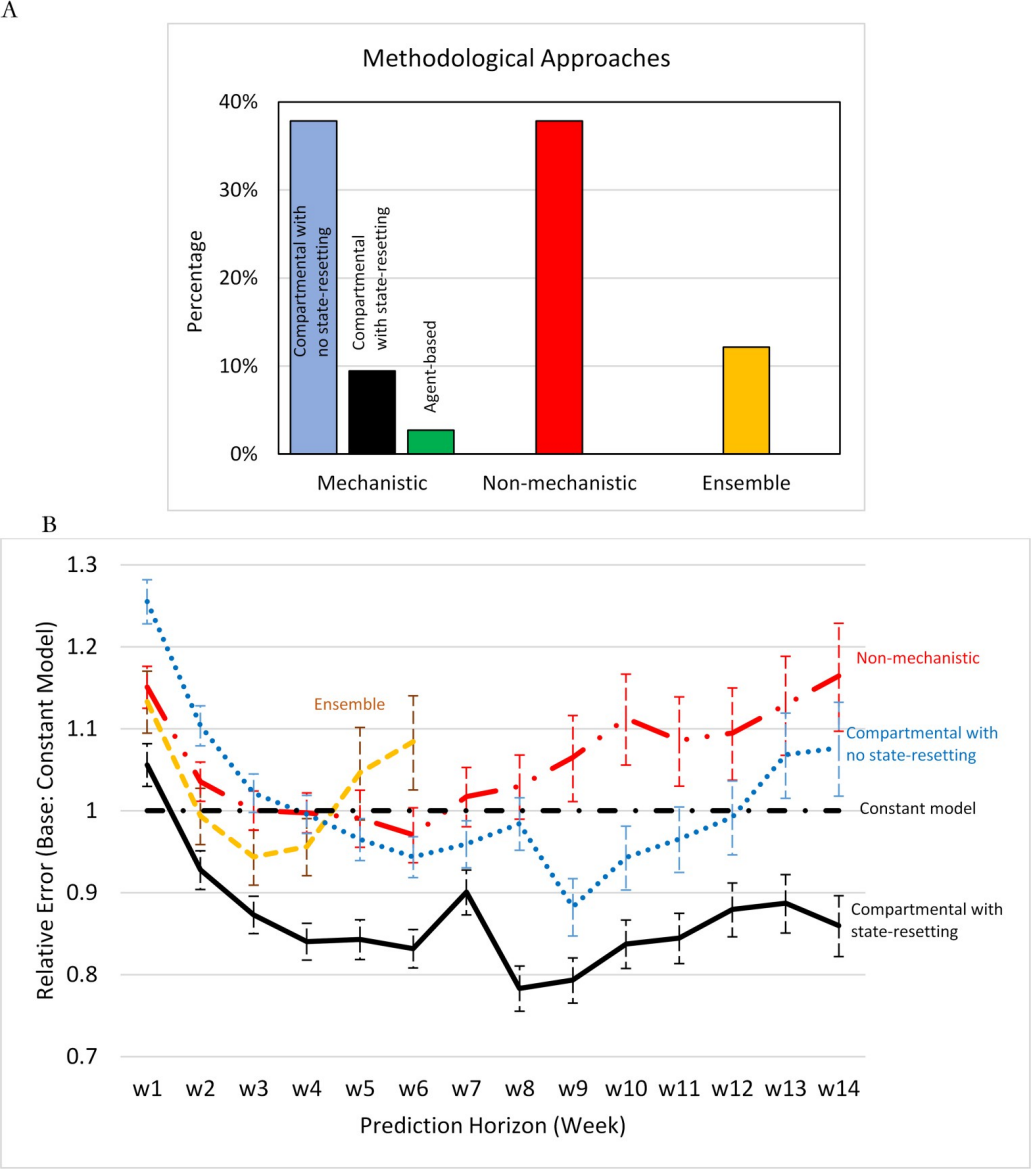
A) Methodological approaches and B) Death projection performance of the CDC model set over different time horizons compared to a constant model.

Models on the CDC Hub (CDC Model Set) varied with respect to several architectural features (Fig 1A; see S1 Text for more details). Of the more than 70 modeling groups listed on the CDC’s website, about half used conventional SEIR-based (mechanistic) architectures. These models are distinguished by their use of variables and mechanisms that mimic the corresponding real-world processes. They varied in their aggregation assumptions, spanning simple SIR (three-compartment) models to those incorporating documented vs. undocumented cases, details of hospitalization and intensive care, disaggregation to the county level or by age, and commuting patterns across different locations. Only two models used individual-level disaggregation. For calibration, most models used death data (for death projections), but many also used case data, and some utilized testing, mobility, and hospitalization data as well.

Mechanistic models endogenously simulate estimates for the measured variables, which could inform both historical comparisons and future projections. They could also reset the state variables in the model at some regular interval to better correspond to measured data. Such “state-resetting” is especially important at the start of making projections because it aligns the state variables in the simulation model with observed history. State-resetting was pursued by 9% of the models, either explicitly (e.g., some using heuristics and some based on more formal methods such as Kalman filtering) or implicitly, when the mechanistic model informed regressions that utilized historical data to estimate the parameters of a dynamic model. Some 15% of models included coupled mechanistic structures for different age groups to capture variations in transmission intensity and fatality, or to represent spatial dynamics of the spread of the disease for different regions. Calibration of mechanistic models to data spanned Bayesian methods, heuristic error terms, and machine learning approaches.

About 38% of modeling groups relied on models that lacked a mechanistic representation of the spread of an epidemic; we label these as non-mechanistic models that incorporated different curve-fitting approaches such as time-series models to fit a function of time to the daily cases or deaths; linear or non-linear regression models, which could include regional variables, temperature, and COVID-related information on a daily basis; and non-parametric machine learning models. About 12% of the modeling groups employed ensemble models combining results of several non-mechanistic and/or mechanistic models. Some of the teams changed their modeling approach overtime, in those cases our analysis considers their most recent documented approach.

Next, we focused on the 61 models that offered death projections, and systematically investigated the associations between their forecast accuracy and their methodological approach using regressions. We used the absolute prediction error normalized by a location’s (state or national) population as the primary measure of prediction accuracy, and compared each model type in Fig 1A with a constant model, using linear regressions with location-forecast date-forecast horizon fixed effects (see details in S2 Text). Here a constant model is a simple predicator that projects all future deaths to be the same as the deaths last observed. On average, forecast accuracy deteriorated by 8% for each weeklong extension of the forecast horizon, but there was considerable heterogeneity in deterioration rates across models. Fig 1B summarizes the results of regressions.

In the short term, non-mechanistic and ensemble models performed better than compartmental models that do not benefit from state-resetting; but the orders reversed beyond 4–5 weeks of projection horizon. Many models outperformed the constant model in the mid- to long term, and compartmental models with state-resetting outperformed, on average, all others in both the short- and long term. The differences were statistically significant (see S2 Text for full regression reports). Additional analysis suggests the relative advantage of mechanistic models may grow more quickly with forecast horizon when death rates change direction (i.e., after a peak or a dip; see S5 Text).

These results may be intuitive: mechanistic compartmental models use structural features of the phenomenon to zero in on the more promising regions of model specification space; absent those constraints the space of possible models is extremely large. While the flexibility afforded by non-mechanistic models may enable excellent fit to historical data and good short-term predictions, predicting the longer-term dynamics becomes increasingly difficult and prone to the risk of overfitting. State-resetting enhances predictions in compartmental models by reducing the gap between model and data due to the accumulation of errors (between model and reality) prior to projection start (though it may also signal more sophisticated modeling approaches). We therefore hypothesize that the use of compartmental models and state-resetting enhance the quality of longer-term predictions.

Note that while there were significant differences in models’ forecast accuracy across these broad categories of methodological approaches, there was much variation left unexplained within each category (see details in S2 Text). Thus, we conducted a further qualitative review of the more accurate mechanistic models (see S1 Text for details). Specifically, a key issue in projection is how transmission rates (e.g. effective reproduction number) change in coming weeks in light of the changing policies and individual behaviors. Several models with good prediction performance leveraged data on government policies and mobility patterns to fit historical transmission rates (e.g., the “YYG-ParamSearch” model). They then extrapolated future transmission in different ways, such as incorporating a weather impact, assuming continued use of existing Non-Pharmaceutical Interventions (NPIs), or reversion to some underlying trend (e.g., a reproduction number of 1 that stabilizes cases) among others [24]. One model (“IHME-CurveFit”) went further and, in its “most likely” scenario, turned policy responses on/off when projected daily death rates crossed 8 deaths/million/day in a locale [24] (this model started non-mechanistic but changed to a mechanistic one early in the study period). These approaches generally recognized that NPIs play a major role in regulating transmission rates, informing our third hypothesis: accounting for changes in behaviors and policies in responses to the evolving state of the epidemic enhances forecast accuracy. However, the CDC model set offers no standard way to capture these changes in human responses.

## 3. Second study: Designing a model to assess prediction-enhancing features

While study one offers some intriguing hypotheses about the promising features of model architectures, those insights are based on associations and may not be causal. Thus, in study two we take the next step towards systematically analyzing the effectiveness of those promising features by developing a simple model that only includes those features and allows for assessing their predictive value. Key to the proposed model is an endogenous formulation of transmission rates: we formulate NPIs as a continuous function of evolving states of the system, specifically, the recently perceived risk of death. This formulation parallels the existing models, connecting economics and epidemiology [25,26] but using a more behavioral (rather than rational) response function [18,27].

### 3.1. Methods

Study 1 highlighted three features as promising for long-term predictions: having mechanistic models, using state resetting, and capturing impact of behavioral responses to changing risks. To test these hypotheses and assess the practical value of each feature, we design a simple predictive model that incorporates the promising features: it is mechanistic (following the SEIR framework); it uses state-resetting before projection; and it incorporates the behavioral feedback loops, where NPIs and thus transmission rates change endogenously in response to the perceived risk of death. We also include in this model a seasonality factor. Prior research shows a modest reduction in transmission rates in warmer days and more minor impacts of ultraviolet index and humidity [28,29]. Noting that such seasonality effects are captured among better predictive models in the CDC set, we also include input for the impact of weather on transmission rates. For this purpose, we use only inputs that were publicly available from the beginning of the prediction period (i.e., since May 2020) [30] and thus are usable in fair comparisons across models. We call the resulting model SEIRb to highlight the importance of endogenous *behavioral* responses to risk (a feedback loop, rather than a new state, thus lower case ‘b’; see explanation below and S4 Text). We then compare the predictive power of this model and its variants that omit each feature—state resetting (-NoRst), behavioral response (-NoB), and weather effect on transmission (-NoW)—to quantify the importance for prediction accuracy of each feature. We also compare the baseline model with existing models on the CDC forecast hub to inform the overall value of such a simple model in comparison with a host of alternatives.

SEIRb excludes many other promising features and is not fine-tuned for this forecasting task; as such, it is indicative of what can be achieved with only the hypothesized features, but leaves much room for improvement. For example: we keep the model very simple (only the four states of Susceptible, Exposed, Infectious, and Removed are modeled for each U.S. state/territory), and ignore loss of immunity, vaccination, variants, medications, differences in acuity, symptoms, hospitalization, age distribution, travel networks, or disaggregation to county level. Further, while more sophisticated methods (such as Kalman and Particle filtering) exist, we heuristically reset state variables once just before the start of projection. In addition, we only use data on deaths and cases, ignoring data on testing, hospitalization, age distribution of cases, and mobility patterns, among others. Calibration is used to find a handful of model parameters for each state and estimation/projection date independent of findings from other locations or projection dates. Finally, we do not fine-tune the uncalibrated parameters of the model (e.g., disease duration), or the model structure, to enhance predictive power, nor do we change model structure across states or over time (e.g. to capture changes over time in fatality rates, or emergence of Alpha variant). S4 Text provides a more complete listing of simplifications of SEIRb. In short, SEIRb is very simple, even simplistic.

#### Model Structure

With these guidelines in mind, we begin with the basic SEIR model and add to it a seasonality factor (w) from prior literature [30] and the behavioral response feedback. This feedback reflects how transmission intensity (*β*) responds endogenously to perceived risk (*f’*: a first order lag of per capita death rate) and includes two free parameters *α* and *γ*:

$$\beta =\beta_{0}w\frac{1}{{\left(1+\alpha f\prime \right)}^{\gamma }}$$

**Fig 2:**
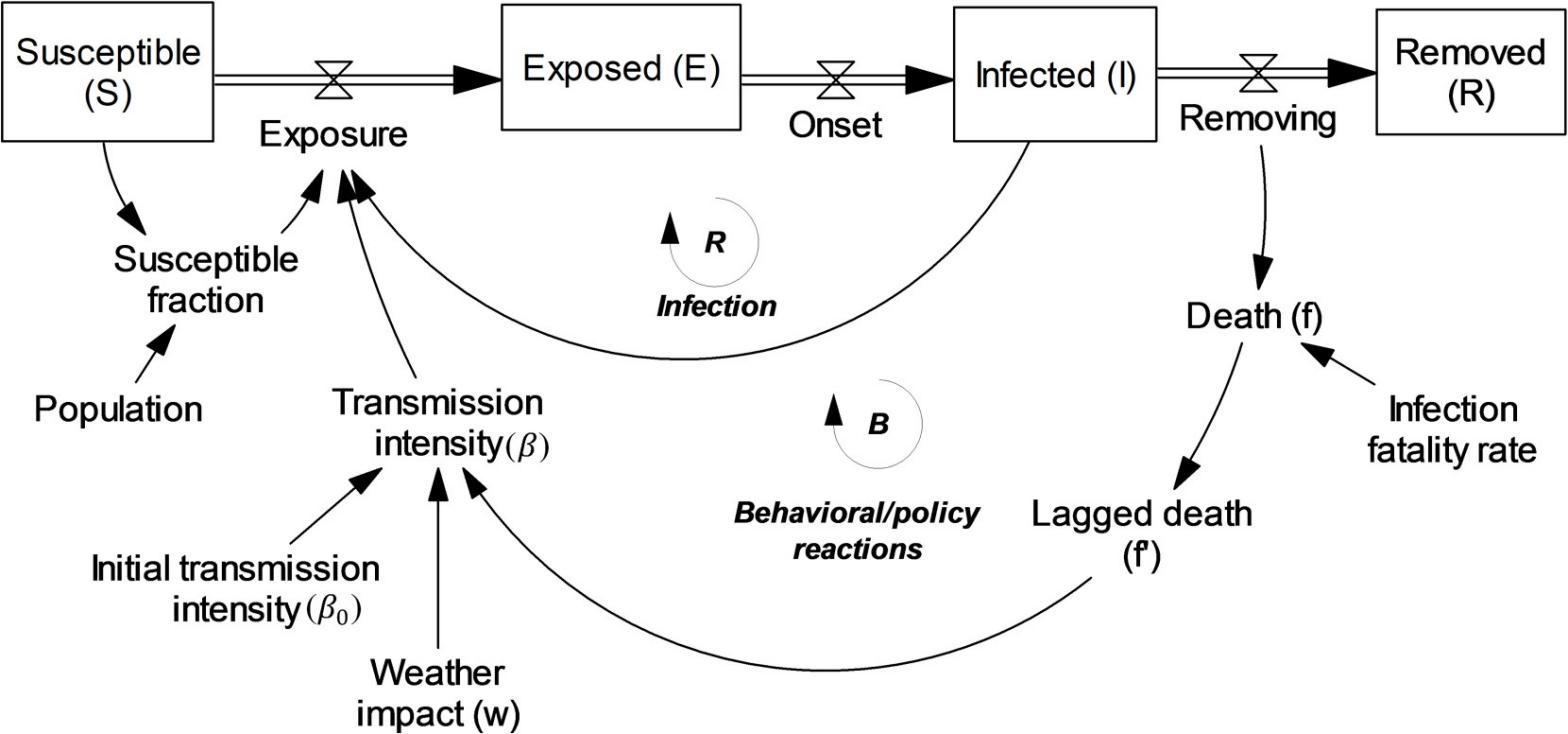
A conceptual representation of SEIRb. Boxes represent state variables (stocks/compartments) while double-lined arrows with valve sign represent flows between states.

We include two lag times for upward and downward adjustment of perceived risk, reflecting the possibility that individuals and governments respond to increasing risk levels more quickly than they abandon precautions when deaths go down. These lag times, along with *β*_0_, *α, γ*, and the infection fatality rate, are the primary parameters we estimate for each location and calibration date. Fig 2 is a graphical overview of the model and full equation listing is in S4 Text.

#### State Resetting

Typical (i.e., “pure”) simulation models use only the initial values of state variables (i.e., the S, E, I, and R in our model) and model formulations to simulate future trajectories (in this case deterministically). However, reported infections and deaths are informative about the current values of those state variables. In fact, one could adjust the state variables over time based on observed data (e.g., begin with pure simulation results and shift values toward those indicated by data). This is the basic idea of state-resetting that is used, implicitly or explicitly, in some of the predictive models. Here we pursue state-resetting only once at the end of the estimation period, when the state variables for exposed and infected are reset to values consistent with the latest death data and death and case trends available in recent history (see S4 Text for details). More sophisticated approaches, such as Kalman and Particle filtering, exist for state-resetting, but we do not apply those in the interest of simplicity.

#### Calibration and Projection

We calibrate the SEIRb (and its variants) with data available until each projection date for each location included in Study 1, and make predictions for the next 20 weeks. Thus SEIRb predictions are directly comparable for those available from the CDC hub and used in Study 1. We then compare forecast errors with predictions across CDC models with those from SEIRb. Estimation is pursued through maximum likelihood, using a Negative Binomial likelihood function to match data and simulations for daily deaths and cases. The model does not project cases in the future, but only uses case data to find more accurate estimates of historical transmission intensity, which then inform the estimation of the behavioral response function.

### 3.2. Results

**Fig 3:**
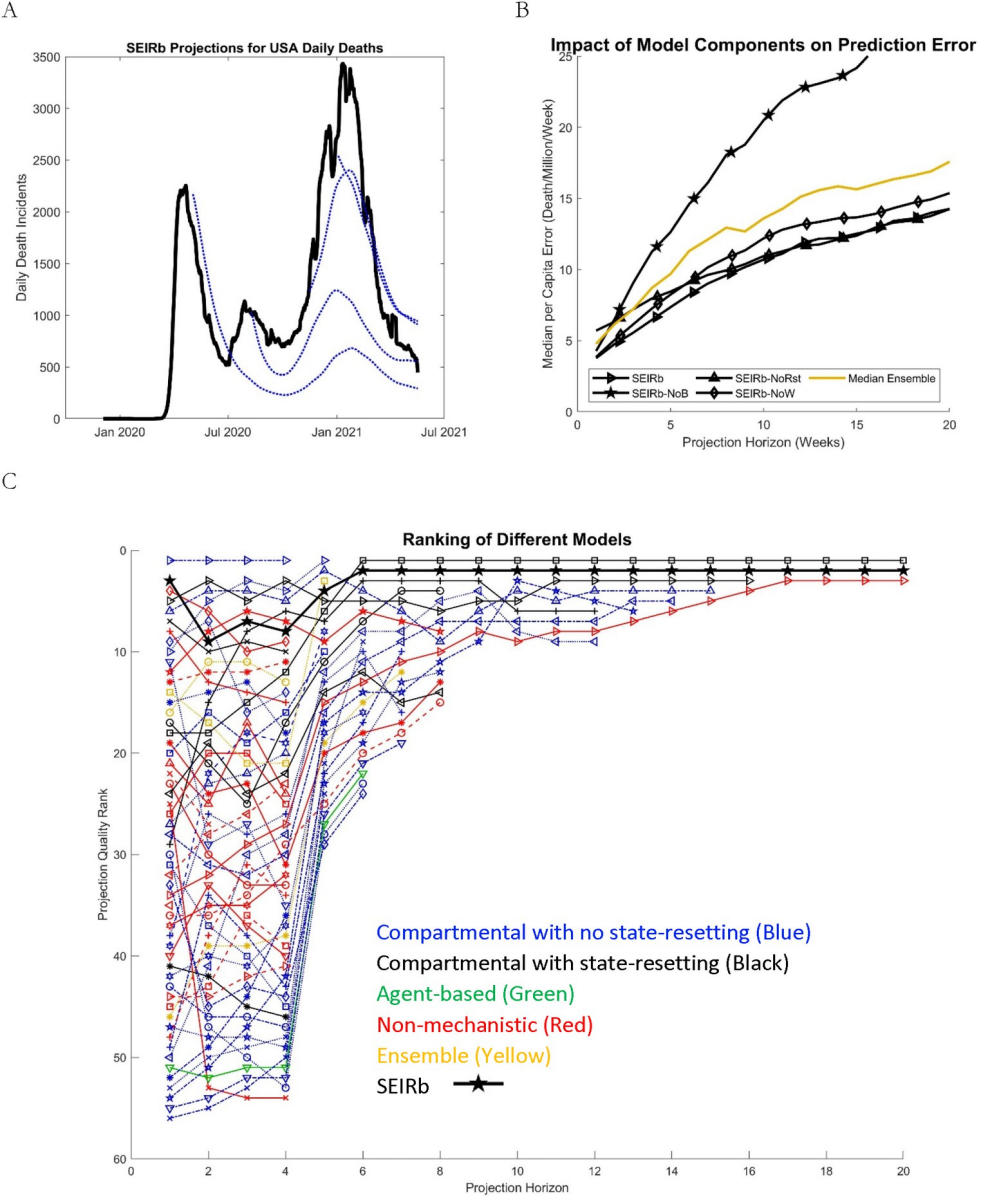
Comparison of forecasting quality among different models. A) Data (solid-black) and predictions (dotted lines) for SEIRb national forecasts made based on data by 5/2/20, 8/8/20, 11/14/20, and 1/2/21. B) Forecasting error per capita for SEIRb and its variants (without seasonality: -NoW; without behavioral feedback: -NoB; without resetting: -NoRst) compared with the median ensemble forecast from the CDC model set and the SEIRb group. C) Forecast quality ranks for the CDC model set and the SEIRb group, based on regressing Ln (per-capita projection error) against models, controlling for location-horizon-week combinations. Individual model names corresponding to each line are available in Fig C in S5 Text. Color codes: compartmental models without state-resetting (blue); with state-resetting (black); non-mechanistic (red); agent-based (green); and ensemble (yellow).

Fig 3 shows the Study 2 results. Panel A shows a sample of national predictions of SEIRb (obtained by summing across all state projections; we don’t estimate a separate national model), starting from five different projection times. In panel B, we compare the predictive power of different variants of the SEIRb model. Every Sunday from 5/3/2020 until 3/13/2021 (i.e., 46 projection dates), each model, calibrated to data until the previous day, provides 1 to 20-week-ahead weekly death projections for all U.S. states and territories with populations greater than 200,000 (53 locations). We compare these projections with actual data for deaths for each location through 5/17/2021, a total of 46,116 comparisons for each model.

The performance of a median ensemble (of the 61 models of the CDC hub plus the SEIRb set) is also plotted for comparison. Median ensemble is a promising approach to improve predictions: by incorporating information from all predictions from a group of models, while excluding outliers, it significantly improves predictions over any typical model [20,31,32], and thus it is a tough standard to beat. SEIRb outperforms the median ensemble. Comparisons of per-capita prediction errors across models show that: 1) endogenous behavioral response is central to the predictive value of the model. Absent this feature, the simple SEIR model significantly underperforms the median ensemble (and most of the individual models in CDC set); 2) state-resetting provides notable value in the short-run, but not beyond 8-weeks ahead horizons; and 3) incorporating seasonality effects adds value, especially over longer prediction horizons. Despite the overall quality of median ensemble forecasts, variants of SEIRb outperform this benchmark over longer horizons as long as a behavioral response feedback loop is included.

In panel C, we compare SEIRb against all models in the CDC group that include at least 50 death predictions for a given projection horizon. We rank the models based on each one’s fixed effect in regressing the (log of) per-capita absolute error (of death predictions). Not every model provides projections for every location, week, or horizon. Thus, a fair comparison across models requires controlling for idiosyncratic challenges of forecasting any specific location, on a specific date, and for a given horizon. We include a regression control for each location-horizon-projection date combination, thus ensuring fair comparisons across models. That said, our results are qualitatively robust to alternative performance measures such as head-to-head win fraction and errors normalized against constant model (those are reported in S3 Text, along with raw population-normalized errors).

About half of the models halt projections at a 4-week horizon, making it impossible to include their longer-term performance in comparisons. Using this measure, in the short-run the models “UMass-MechBayes”, “YYG-ParamSearch,” “USC-SI-kJalpha,” and “Karlen-pypm” are among the top performers, while SEIRb remains in the top 10. Top-performing longer-term projections are offered by “IHME-CurveFit” and “YYG-ParamSearch,” both mechanistic models that include state-resetting, with SEIRb offering the second-best performance for horizons greater than 5 weeks. Only a handful of models have consistently submitted very long-term (>12 weeks) projections, so those rankings are not particularly informative; yet comparisons against the constant model (see S3 Text) show that SEIRb predictions remains informative all the way through 20-week ahead. Note that our focus in this paper is not on comparing individual models in the CDC set, so we only show the individual model names in Fig C in S5 Text.

## 4. Discussion

Forecasting over long horizons is difficult, and yet extended forecasts, whether from implicit mental or explicit formal models, are indispensable for many individual and policy choices. Epidemics are complex forecasting problems [15,16]: they include reinforcing contagion mechanisms that create exponential growth, are moderated by randomness in environmental and social determinants of transmission, and are subject to endogenous human responses to evolving risk perceptions. The high stakes of forecasting during the COVID-19 pandemic have created a unique opportunity to better understand how we can enhance forecasts of contagion dynamics. Correlating the features of the CDC hub’s predictive models for COVID-19 deaths in the United States against their forecast accuracy, we propose that mechanistic models that reset state variables at projection time are promising for epidemic forecasting. Critical to the performance of models is how they predict changes in behaviors and NPIs that condition transmission rates. Furthermore, including an impact of seasonality on virus transmission can improve COVID-19 projections. We provide evidence in support of these hypotheses by building a simple model that incorporates (only) these features and offers predictions on par with the best models in the CDC set.

Among these features, capturing the endogenous changes in transmission rates offers the greatest benefit (see Fig 3B), and is arguably the least appreciated among the models we reviewed. While several approaches have been used to predict future changes in transmission rates exogenously, we could not establish that any other models—except for one model in the CDC set [24]—had captured this feedback process endogenously. This observation is somewhat surprising, as the importance of behavioral feedbacks in the dynamics of contagion are known both from historical analysis [33] and the modeling literature [18,34,35], and has been supported by the recent research on COVID-19 modeling [18]. In fact, that feedback provides the primary path to replicating the observed waves of the epidemic (without resorting to exogenous drivers such as weather or holidays): a rise in deaths elevates risk perception, strengthens NPIs and compliance [33], and thus brings down cases until the now-reduced death rate diminishes perceived risk, relaxing NPIs and compliance and launching the next wave. Overall, endogenizing behavioral responses to perceived risk points to a significant opportunity for enhancing predictive models in the context of epidemics, as well as for designing more effective policies/interventions that go beyond the assumptions of exogenous and static individual behaviors [32].

The behavioral response feedback also informs the surprisingly good performance of a naïve, constant model that outperforms many models in the CDC set over different forecast horizons (See Fig 1B and S3 Text). Specifically, cases will grow in a community until this feedback brings down transmission rates and cuts the effective reproduction number to below 1. A reproduction number well below 1, however, will not be sustained for long: dwindling cases will alleviate risk perception and increase community interactions, raising transmissions and the reproduction number. So, this feedback process creates a natural attractor for the system at a reproduction number of 1, which has also been empirically demonstrated [18]. The key insight is that a reproduction number of 1 entails limited change in the number of cases and (assuming constant IFR) deaths in the coming weeks. That is exactly the prediction we used for the naïve, constant model. Thus, the constant model accounts for an important implication of the behavioral response mechanism and, as such, may perform better than one might expect from a simple heuristic.

Are long-term forecasts of epidemics uninformative, as some have suggested [16,17]? Answering this question requires settling on a benchmark for what constitutes an informative forecast. For example, we find that the gap in predictive power across models expands as we look at longer horizons (see S3 Text). Thus, assuming the benchmark of a *typical* model, one could argue that predictions from more accurate models become *more* informative over longer horizons. However, while the best-performing models beat the constant model across all horizons, the gap between the two does not increase beyond 12-week horizons, suggesting an upper bound on the value of long-term predictions beyond a cognitively simple and theoretically plausible forecast heuristic. Nevertheless, we also find no evidence to suggest a decline in the informativeness of (good) forecasts within a 20-week horizon.

Our results should be interpreted in light of a few caveats. First, epidemics across U.S. states have been interdependent and, therefore, prediction performances are correlated: a model that gets the Fall 2020 peak right may do well in our comparisons, but that may be due more to luck than to an intrinsically valuable feature of that particular model. Indeed, we noted that comparisons of predictive power over dozens of data points, or data from single locations or limited time windows, could be misleading. One needs thousands of test data points to draw reliable conclusions. Second, comparisons using other measures could offer different insights; for example, prediction confidence intervals are informative about features of models related to accurate quantification of uncertainty in predictions [19]. Third, we used projections from before April 2021, when vaccinations and Delta and Omicron variants had not yet changed death trajectories qualitatively; SEIRb may well need additional details to account for those factors to continue offering quality forecasts. The emergence of variants also highlights another unpredictable element that limits the usefulness of long-term forecasts. Fourth, we have not assessed projections for cases, a task that may require more complex models due to variable ascertainment rates across time and states. Fifth, SEIRb was made after the fact, even though we designed fair comparisons, our model has probably benefited from the more recent knowledge of the pandemic as well as various hindsight biases of which the authors may not be aware. It also excludes many features that have been discussed in the literature [32] and could be valuable in forecasting performance (see S4 Text). As such, its primary role is not in offering real-time predictions of COVID deaths.

Instead, SEIRb offers a relatively reliable ‘starting point’ for epidemiological modeling. Such small, feedback-rich models offer clarity on what matters and help communicate broad dynamic insights [36]. With SEIRb we take a step towards the grand challenge of modeling human behavior and its impact on the progression of an epidemic [19]. We also recognize that other behavioral feedbacks could be valuable additions. For example, overtime IFR has declined due to factors such as improving patient care and differential social distancing among the youth vs. the elderly. Future models could add such experiential learning processes by formulating IFR to be inversely related to past cumulative cases or deaths.

Extrapolating from these findings, forecasting models responding to future epidemics would benefit from starting small, incorporating key mechanistic features and important behavioral feedbacks, and incorporating simple state-resetting approaches. The models could then be expanded to account for important novel features identified by emerging empirical research in the evolving epidemic. While these results are most informative for epidemic forecasting, one may speculate about other forecasting challenges. For instance, we may expect that bringing together the physics of a problem with behaviorally realistic representations of human decision making create feedback mechanisms with insights for predictive models beyond epidemics. For example, climate risks have been shifting R&D, consumption and investment patterns which regulate future emissions and risks; or falling infant mortality has contributed to reduced reproduction rates which enhance healthcare coverage for new infants. More generally, from climate change and demographic transitions to economic planning, feedback processes that cross physical and biological systems and human choices are regulating long-term dynamics. From United Nations to various national agencies, modelers offer long-term projections (sometimes over many decades) crucial to everyday managerial and policy choices. Predictive as well as policy models that explicitly capture such feedbacks (rather than using exogenous predictions from one model to drive the other) may offer promising avenues to enhance longer-term predictions and policy robustness.

## Supporting information

S1 Text Coding the CDC hub models. Details of the coding for CDC hub models and some key features are explained.
(DOCX)

S2 Text Details of Statistical Analyses. Details of the statistical analysis comparing CDC hub models are explained. **Table A.** Regression Results comparing CDC model performance to a constant model. **Table B.** Regression Results comparing CDC model performance to a constant model, controlling for model documentation and affiliation.
(DOCX)

S3 Text Comparison of models based on other measures. The performance of SEIR-b models are compared with CDC hub models based on a variety of alternative metrics. **Fig A.** Comparison of performance of models based on head-to-head win fraction. **Fig B.** Comparison of performance of models based on normalized error relative to constant model. **Fig C.** Comparison of performance of models based on population-normalized absolute error
(DOCX)

S4 Text Model Documentation. The details of simulation model formulation, calibration and improvement opportunities are explained. **Table A.** List of parameters estimated in model calibration and their search (feasible) ranges.
(DOCX)

S5 Text Additional Analyses on Model Performance. Models’ performance using alternative metrics as well as by different periods are explored. **Fig A.** Death projection performance of the CDC model set over different time horizons compared to a constant model based on Interval Score. **Fig B.** Death projection performance of the CDC model set over different time horizons compared to a constant model before turning points (a) and after turning points (b). **Fig C.** Forecast quality ranks for CDC model set and SEIRb (a) pre- and (b) post- turning points.
(DOCX)
