# Supplementary material for: Enhancing long-term forecasting: Learning from COVID-19 models
Source: PLoS Comput Biol. 2022 May 19;18(5):e1010100. doi: 10.1371/journal.pcbi.1010100 (PMC9119494; doi:10.1371/journal.pcbi.1010100)
Supplement: S1 Text — Details of the coding for CDC hub models and some key features are explained. (DOCX) [file pcbi.1010100.s001.docx]

## S1 Text: Coding the CDC hub models

## Coding details

A primary list of models that contributed to the CDC Covid forecast hub was obtained from CDC’s website (<https://www.cdc.gov/coronavirus/2019-ncov/cases-updates/forecasts-cases.html>). The list was verified by comparing with two pre-print manuscripts on medRxiv co-authored by the contributors to the hub ([38, 39]). The list was then compared with available forecast data on the hub, and as a result five missing models were added to the list, yielding a total of 74 models. We then narrowed down the list to the models that provided COVID-19 death forecast, which included a large majority of the models (n=61). The final list of models included:

AIpert-pwllnod, BPagano-RtDriven, Caltech-CS156, CEID-Walk, CMU-TimeSeries, Columbia_UNC-SurvCon, Covid19Sim-Simulator, CovidActNow-SEIR_CAN, CovidAnalytics-DELPHI, COVIDhub-ensemble, CU-select, DDS-NBDS, epiforecasts-ensemble1, Geneva-DetGrowth, Google_Harvard-CPF, GT_CHHS-COVID19, GT-DeepCOVID, IEM_MED-CovidProject, IHME-CurveFit, IowaStateLW-STEM, IUPUI-HkPrMobiDyR, JCB-PRM, JHU_CSSE-DECOM, JHU_IDD-CovidSP, JHUAPL-Bucky, Karlen-pypm, LANL-GrowthRate, LNQ-ens1, Microsoft-DeepSTIA, MIT_CritData-GBCF, MIT_ISOLAT-Mixtures, MITCovAlliance-SIR, MOBS-GLEAM_COVID, MSRA-DeepST, NotreDame-FRED, NotreDame-mobility, OliverWyman-Navigator, PSI-DRAFT, QJHong-Encounter, RobertWalraven-ESG, RPI_UW-Mob_Collision, SigSci-TS, SteveMcConnell-CovidComplete, STH-3PU, SWC-TerminusCM, TTU-squider, UA-EpiCovDA, UChicagoCHATTOPADHYAY-UnIT, UChicago-CovidIL, UCLA-SuEIR, UCM_MESALab-FoGSEIR, UCSB-ACTS, UCSD_NEU-DeepGLEAM, UMass-MechBayes, UMich-RidgeTfReg, UpstateSU-GRU, USACE-ERDC_SEIR, USC-SI_kJalpha, UT-Mobility, Wadhwani_AI-BayesOpt, and YYG-ParamSearch.

Two researchers (NG and RX) analyzed the models based on any available information, and coded their methodological approaches. Specifically detailed notes were taken about modeling approaches based on documentations on websites, related journal publications, and in a few cases upon contacting modelers with clarifying questions. Other important sources of information included a webpage (<https://zoltardata.com/project/44/forecasts>) which includes self-reported brief information (about 1-2 paragraphs) on methodological approaches of each model. This website particularly helped with several models that lacked any further technical documentation. We also consulted the information on a related GitHub repository (<https://github.com/cdcepi/COVID-19-Forecasts/blob/master/COVID-19_Forecast_Model_Descriptions.md>). We further checked the modelers’ websites, blogs, or twitter links, for updates, possible changes in methods, or more methodological details. A few groups changed their models throughout the forecast for which we considered their most recent approach in our coding.

The primary coding question was related to the modeling approach. Initially five mutually exclusive and exhaustive groups of modeling approaches were identified and used to categorize the models:

1. Mechanistic compartmental models: this is the conventional approach in epidemiology to model the spread of an infectious disease in which the population is represented by different compartments. A common example is the Susceptible-Exposed-Infected-Removed (SEIR) model.
2. Non-mechanistic models: these models do not capture the physics of the spread of the disease, and instead, by using different data-analytic approaches, try to uncover association between death incidents and other variables. These models include different forms of parametric and non-parametric regression models and machine learning techniques.
3. Ensemble models: these models provide estimates based on combining two or more distinct models’ (which could be each mechanistic and non-mechanistic) forecasts.
4. Others: Only two models that used agent-based modeling approaches belonged to the “others” group. We thus name the group agent-based models. These models are mechanistic and representing individuals explicitly rather than lumping them together in mixed compartments.

Among the first group of mechanistic compartmental models, we further categorized them into mechanistic models with state-resetting vs. mechanistic models without state-resetting based on how simulation outcomes were combined with the data. A sub-group of mechanistic models used state-resetting procedures to improve their forecast accuracy. Simply put state-resetting is a procedure to combine simulation outcomes from the model with observed data to come up with more accurate values for the state variables in the model, and then to reset the state variables to those more likely values which would potentially enhance the quality of both parameter estimation and predictions. An example of state-resetting is an SIR-based model that periodically estimates the number of active cases from the case data, feeds it to the “I” variable, and then simulates the model for the purpose of projection. In this example one could also use a backward estimation of active cases based on reported death. In fact a more sophisticated method could combine both estimates into a better estimate for “I.” While explicit and structured methods for state-resetting, such as Kalman and Particle filtering, are well known, for simpler models one can use simple heuristic resetting with much lower computational costs. Moreover, a few models did state-resetting implicitly. For example, they estimated a regression model that was based on an SEIR-type mechanistic formulation. Such regressions would calculate the state variables based on observed data every period and as such are doing state resetting implicitly.

We further examined models’ structures looking into methodological details. A major challenge however was the large variation of the quality of documentation of the models. While some of the models had reported sufficient details for replication of their models and findings, others may only had short documents, or a few lines of explanations about the underlying models and estimation techniques. Nevertheless we coded the models based on:

- data inputs:
  - variable type (e.g., death data, case data, hospitalized data);
  - approach to use data: data as an exogenous input vs. data for model calibration;
- output variable:
  - the main predictions of the models (e.g., death, case);
  - the time horizon of the predictions;
- approach to estimate transmission intensity:
  - is transmission intensity constant or changing;
  - are they modeling social distancing explicitly or implicitly, and if so how;
- approach to project future trajectory of transmission intensity:
  - are they assuming transmission intensity (and the reproduction number) is going to stay constant, or change;
  - if changes, do they model change in transmission intensity (and the reproduction number), or only do scenario analysis (constant varying transmission intensity);
  - if they model change in transmission intensity (and the reproduction number), does it include an endogenous mechanism or it is an exogenous time series based on expected time to reopen;
- modeling mobility:
  - are they modeling change in mobility;
  - are they using mobility data;
- General information such as:
  - modelers’ affiliation (academic or non-academic);
  - disciplinary background; and
  - availability of technical documentation.

Furthermore, for different methodological approaches we specifically looked for the following criteria:

- For mechanistic models with adequate documentation:
  - details of the compartmental structure: compartments (Simple SIR vs. SEIR vs. more compartments for capturing different stages of illness and symptoms);
  - do they include coupled age-structure;
  - do they have coupled compartments with commuting across regions;
  - parameter estimation (model calibration):
    - sources of parameter values;
    - do they calibrate their model with the data, and if so what is the payoff function and methods to find optimal parameters;
  - weather impact:
    - do they include any estimate of weather impact on transmission intensity or the reproduction number;
- For non-mechanistic models with adequate documentation:
  - Specifics of the method:
    - From simple regression models to more sophisticated curve-fitting approaches and machine learning techniques;
  - weather impact:
    - do they include any estimate of weather impact in their model;
- For ensemble models:
  - - the type of models used in the ensemble.

Moreover, we made note of any interesting observation such as change in method of forecast and models or change in parameter values or attempts for fine-tunings.

After NG and RX independently coded the models, they shared and discussed their results. The initial inter-rater reliability (percent agreement between the two raters) was 90%, high enough that did not require any changes in the coding process. The coders converged on the final results after a discussion and those results inform the relevant regressions. All three authors discussed major lessons learned through reading the documents.

## Common features of the models

A few initial observations were noteworthy for the research team:

1. **Only two (<4%) models used agent-based architectures.** In contrast to our initial expectation, only 2 models used agent-based individual-level approaches, and they seemed to have stopped projecting after a few rounds. Only one of them provided death projection. On the other hand, the majority of the models preferred to model at US state- or county-levels, using compartmental or non-mechanistic approaches. Lack of ABM approaches may partially be explained by the computational costs of these methods in light of the calibration requirements and large parameters spaces they typically include.
2. **About 38% of the models used non-mechanistic approaches**. With the growing attention to data-driven methods across various fields we observed a considerable number of non-mechanistic models. Particularly about 16% of the models used machine-learning techniques for projection confirming a growing trend in the application of AI. Many of these models were developed by computer science and engineering researchers.
3. **About half of the groups used conventional SIR-like models with modest modifications**. Given the growing alternatives for modeling the dynamics of transmission it was interesting that still many modelers start with the classical architectures. The prevalence of S(E)IR models, some including more details about asymptomatic cases or hospitalized cases, and a few using detailed coupled compartmental structures where people travel between different regions puts these methods at the heart of the existing approaches.
4. **Among mechanistic models, the majority used simple techniques for parameter estimation.** Most mechanistic models tried to utilize recent documented measures about COVID-19 (such as infection fatality rate or the disease duration) from other research publications. They then estimated a few unknown parameters such as the basic reproductive number (or transmission intensity), often by fitting the simulation with data in a nonlinear optimization. The process of parameter estimation was often simple, minimizing the mean square error between simulation and data on daily or weekly deaths/cases. The search strategy for optimal parameters ranged from simple algorithms to more advanced machine-learning techniques. Only a handful of groups used more sophisticated estimation approaches with explicit likelihood functions and state-resetting (e.g. Markov Chain Monte Carlo simulations and Kalman Filtering).
5. **For fitting simulation with the historical trends, mechanistic models commonly considered non-constant reproductive numbers.** A large number of models tried to incorporate change in the reproductive number (or transmission intensity of ß). Some of them used different mobility data, and estimated change in transmission intensity as a function of change in mobility. Others used estimation of the reproductive number from daily cases. A few groups used data on when each US state started their social distancing policies. Such data were fed into the model to better estimate change in the reproductive number. For example, a few models assumed a specific percentage decline in the reproductive number after implementation of lockdown policies.
6. **For the purpose of projection, mechanistic models commonly assumed constant reproductive numbers.** Most models lacked techniques of projecting the reproductive number (or transmission intensity of ß). A large majority used their latest estimate of the reproductive number from the past data for projecting the future cases.
7. **Modelers updated their models through the course of the pandemic.** Like any other social setting, modelers learned from the past projections and tried to incorporate new ideas to improve their future projections. Several of them updated their parameter values as more data became available about the nature of the disease. A few groups dropped out after a few projections, and a few others joined the hub several months after the starting date. We noted that a few groups changed their modeling approaches too. The common direction of changing modeling methods was from curve-fitting to mechanistic compartmental models.

## Features of top performing models

Our primary analysis uses consistent coding applicable across all models. Given the significant heterogeneity in the documentation of CDC model set this analysis does not inform more detailed features of models beyond a few aggregate categories. We therefore studied the top 3 models in the long-term prediction performance in more depth (short-hands: IHME, YYG, BPagano) to learn about more specific features that might have improved performance beyond those measurable across all models. It is important to note that the IHME model of COVID-19 pandemic started as a non-mechanistic model but moved away from their initial curve-fitting approach towards a detailed, mechanistic model, which offered substantially better predictions than their curve-fitting approach. Since the first incarnation of the model was non-mechanistic, many may not have realized this important change.

Importantly, we noted that the assumption of constant vs. changing reproductive number is essential in long-term projections. Among mechanistic models the challenge of modeling a pandemic primarily boils down to the prediction of societal reactions and policy decisions. Two particular models of YYG and IHME are good examples: the former used available reports on states’ plans for opening and the modelers’ best judgment for extrapolating those in future. Specifically, YYG estimated the reproductive number (R(t)) by four main values of (R_0_, R(post-mitigation), R(post-opening), R(equilibrium [sometime after post-opening])=~1), and used a sigmoid function for the transition between R_0_, and R(post-mitigation), and possibly other R-values, where the slope of the function was also estimated through model calibration. The mitigation and opening were based on a New York Times dataset. IHME used a more detailed approach: data on state policies (severe travel restrictions, closing of public educational facilities, closure of nonessential businesses, stay-at-home orders, and restrictions on gathering size) were gathered from press release or state government official orders. The model then estimated the policy effects on mobility and their effect on transmission intensity. In addition, IHME modeled future policy changes endogenously, with a binary feedback mechanism: they assumed that there is a threshold for daily death at 8 per million population, and if simulation forecasts for death pass the threshold, infectivity will decline due to possible implementation of social distancing measures. [24].

Another observation was about state-resetting techniques. For example, in the BPagano the number of daily infections was estimated by shifting daily death backward, and dividing it by the most recent estimate of the infection fatality rate. Then the current active cases (I) was estimated as the sum of daily infections for the duration of the infectious period. The IHME model took a similar approach by using death-based estimation of daily infection as data inputs (rather than simulated outcomes) in the SEIR model.

Some other factors were also noted in the models. High-performing models incorporated the weather effect. IHME for example used flu season as factor in modeling transmission intensity. Moreover, YYG modeled lockdown fatigue which considers that R(post-mitigation) may increase before opening. This model also considered change in infected fatality rate which might be due to healthcare systems’ learning over time or changes in composition of infected towards younger cohorts. Such mechanisms are potentially helpful in better projections.
