# Supplementary material for: Enhancing long-term forecasting: Learning from COVID-19 models
Source: PLoS Comput Biol. 2022 May 19;18(5):e1010100. doi: 10.1371/journal.pcbi.1010100 (PMC9119494; doi:10.1371/journal.pcbi.1010100)
Supplement: S2 Text — Details of the statistical analysis comparing CDC hub models are explained. Table A. Regression Results comparing CDC model performance to a constant model. Table B. Regression Results comparing CDC model performance to a constant model, controlling for model documentation and affiliation. (DOCX) [file pcbi.1010100.s002.docx]

## S2 Text: Details of Statistical Analyses

**Analysis of CDC models**

We used data from the Center for Disease Control (CDC) repository of COVID-19 projections, which included 490,210 point forecasts for weekly death incidents across 57 locations, forecast dates over the span of a year (4/13/2020 to 3/29/2021), 20 forecast horizons (1-week-ahead predictions to 20-week-ahead predictions), and 61 models. We chose the normalized/per-capita absolute prediction error as the basis of comparison for model performance, i.e. the absolute difference between a model’s prediction of weekly incident death and the true weekly incident death, divided by location’s population. As this measure is highly skewed we log-transformed the measure and included 1-99 percentile of the data for further analysis. We further excluded two agent-based models and COVID-hub ensemble (the ensemble of all other eligible CDC models) from the analysis, which resulted in a final sample with 463,305 predictions made by 58 models for each state of the United States, with target end dates ranging from 4/18/2020 to 4/3/2021.

We compared how each type of the model performed in each forecast horizon with a constant model – a model that predicts future weekly incident death to be the same as the weekly incident death last observed. Specifically, we included the weekly incident death predictions from the constant model for each unique combination of location, forecast date and forecast horizon in our sample frame as the baseline, and we included each model type (ensemble, non-mechanistic, mechanistic without state-resetting, and mechanistic with state-resetting) as a key independent variable in a linear regression with location-time fixed effects, and we ran separate regression analyses for each prediction horizon.

As depicted in Table A, the coefficient for each model-type represents the average differences in log-transformed normalized error between that model-type and the baseline (constant) model. Results showed that (1) in one-week-ahead predictions the constant model performs better than all other model types on average (but several individual models outperform the constant one); many models and model types outperform the constant model in mid to long term predictions, with mechanistic model with state-resetting performing the best starting from 2-week-ahead predictions; (2) In short-term non-mechanistic and ensemble models perform better than mechanistic models without state-resetting, but that ordering reversed beyond 4-5 weeks of projection horizon. However, on average mechanistic models with state-resetting outperformed all others in both the short- and the long-term.

As a robustness check we also included more model characteristics, i.e. whether the modelers are affiliated with academia, whether the model has detailed documentation, and the interaction of the two, as covariates and reran the aforementioned analysis (we excluded the constant model from the analysis and used the mechanistic (excluding state-resetting) model as the baseline model). Results are reported in Table B and are consistent with our main results. Interestingly models with documentation performed worse than those without within 1-8 weeks-ahead predictions, but that effect was attenuated (and reversed in 5-8 weeks-ahead predictions) if the modelers were from academia. Note that the within R-squared (excluding the variation accounted for by the fixed effects) were small for all of the models. This is somewhat expected as there was much heterogeneity across model performance [19], and the purpose of this analysis was not to make causal inference about factors influencing model performance but to descriptively compare the model performance based on the broad categories that their methodological approaches fall into. These analyses generate model building hypotheses which we can further test in the simulation. To further investigate model heterogeneity we also conducted more detailed qualitative review for each model in S1 Text.

Table A. Regression Results comparing CDC model performance to a constant model.

|  | (1) | (2) | (3) | (4) | (5) | (6) | (7) |
| --- | --- | --- | --- | --- | --- | --- | --- |
| Outcome:  Log(normalized absolute error) | Week 1 | Week 2 | Week 3 | Week 4 | Week 5 | Week 6 | Week 7 |
|  |  |  |  |  |  |  |  |
| Ensemble | 0.125*** | -0.00640 | -0.0581** | -0.0449* | 0.0457 | 0.0808** |  |
|  | (0.0170) | (0.0176) | (0.0182) | (0.0186) | (0.0275) | (0.0270) |  |
| Non-mechanistic | 0.141*** | 0.0350** | 0.000288 | -0.00241 | -0.00914 | -0.0298 | 0.0170 |
|  | (0.0113) | (0.0118) | (0.0122) | (0.0125) | (0.0179) | (0.0176) | (0.0181) |
| Mechanistic with state-resetting | 0.0544*** | -0.0751*** | -0.136*** | -0.174*** | -0.171*** | -0.184*** | -0.105*** |
|  | (0.0126) | (0.0130) | (0.0134) | (0.0136) | (0.0147) | (0.0143) | (0.0155) |
| Mechanistic (excluding state-resetting) | 0.227*** | 0.0988*** | 0.0214 | -0.00429 | -0.0355** | -0.0580*** | -0.0415** |
|  | (0.0110) | (0.0113) | (0.0116) | (0.0119) | (0.0134) | (0.0135) | (0.0153) |
| Constant | -12.32*** | -12.02*** | -11.81*** | -11.63*** | -11.51*** | -11.37*** | -11.36*** |
|  | (0.00985) | (0.0101) | (0.0104) | (0.0106) | (0.0108) | (0.0105) | (0.0110) |
|  |  |  |  |  |  |  |  |
| Observations | 90,453 | 86,459 | 83,279 | 80,676 | 38,180 | 34,576 | 25,398 |
| Within R-squared | 0.007 | 0.004 | 0.003 | 0.004 | 0.005 | 0.007 | 0.003 |
| Number of location-time combination | 2,847 | 2,790 | 2,738 | 2,681 | 2,623 | 2,566 | 2,331 |

Standard errors in parentheses

*** p<0.001, ** p<0.01, * p<0.05

|  | (1) | (2) | (3) | (4) | (5) | (6) | (7) |
| --- | --- | --- | --- | --- | --- | --- | --- |
| Outcome:  Log(normalized absolute error) | Week 8 | Week 9 | Week 10 | Week 11 | Week 12 | Week 13 | Week 14 |
|  |  |  |  |  |  |  |  |
| Non-mechanistic | 0.0292 | 0.0629* | 0.107*** | 0.0823** | 0.0907*** | 0.122*** | 0.152*** |
|  | (0.0193) | (0.0251) | (0.0255) | (0.0255) | (0.0262) | (0.0273) | (0.0288) |
| Mechanistic with state-resetting | -0.244*** | -0.231*** | -0.177*** | -0.169*** | -0.128*** | -0.120*** | -0.151*** |
|  | (0.0179) | (0.0177) | (0.0179) | (0.0185) | (0.0190) | (0.0205) | (0.0220) |
| Mechanistic (excluding state-resetting) | -0.0158 | -0.125*** | -0.0588** | -0.0352 | -0.00771 | 0.0661** | 0.0737** |
|  | (0.0165) | (0.0202) | (0.0210) | (0.0211) | (0.0231) | (0.0248) | (0.0271) |
| Constant | -11.27*** | -11.23*** | -11.22*** | -11.18*** | -11.12*** | -11.12*** | -11.06*** |
|  | (0.0120) | (0.0123) | (0.0127) | (0.0129) | (0.0134) | (0.0142) | (0.0153) |
|  |  |  |  |  |  |  |  |
| Observations | 21,339 | 13,545 | 12,534 | 11,743 | 10,791 | 9,709 | 8,931 |
| Within R-squared | 0.014 | 0.020 | 0.015 | 0.013 | 0.010 | 0.012 | 0.018 |
| Number of location-time combination | 2,273 | 2,195 | 2,129 | 1,951 | 1,898 | 1,830 | 1,724 |

Standard errors in parentheses

*** p<0.001, ** p<0.01, * p<0.05

Table B. Regression Results comparing CDC model performance to a constant model, controlling for model documentation and affiliation.

|  | (1) | (2) | (3) | (4) | (5) | (6) | (7) |
| --- | --- | --- | --- | --- | --- | --- | --- |
| Outcome:  Log(normalized absolute error) | Week 1 | Week 2 | Week 3 | Week 4 | Week 5 | Week 6 | Week 7 |
|  |  |  |  |  |  |  |  |
| Ensemble | -0.0571*** | -0.0667*** | -0.0464** | -0.0151 | -0.237*** | 0.0167 |  |
|  | (0.0156) | (0.0162) | (0.0168) | (0.0172) | (0.0413) | (0.0456) |  |
| Non-mechanistic | -0.0564*** | -0.0356*** | 0.00495 | 0.0254** | 0.0356* | 0.0342* | 0.107*** |
|  | (0.00766) | (0.00823) | (0.00859) | (0.00879) | (0.0170) | (0.0172) | (0.0200) |
| Mechanistic with state-resetting | -0.217*** | -0.225*** | -0.203*** | -0.212*** | -0.176*** | -0.177*** | -0.0473** |
|  | (0.0103) | (0.0107) | (0.0111) | (0.0113) | (0.0140) | (0.0141) | (0.0161) |
| Academia | 0.00394 | 0.0131 | 0.0204 | 0.0343* | 0.285*** | 0.0728 | 0.125** |
|  | (0.0121) | (0.0126) | (0.0134) | (0.0138) | (0.0372) | (0.0409) | (0.0440) |
| Model documentation | 0.183*** | 0.192*** | 0.175*** | 0.172*** | 0.159*** | 0.195*** | 0.348*** |
|  | (0.0128) | (0.0134) | (0.0138) | (0.0141) | (0.0196) | (0.0194) | (0.0250) |
| Academia*Model documentation | -0.0864*** | -0.121*** | -0.117*** | -0.127*** | -0.553*** | -0.376*** | -0.524*** |
|  | (0.0156) | (0.0162) | (0.0170) | (0.0174) | (0.0400) | (0.0438) | (0.0482) |
| Constant | -12.18*** | -12.00*** | -11.85*** | -11.71*** | -11.51*** | -11.39*** | -11.47*** |
|  | (0.00976) | (0.0102) | (0.0107) | (0.0109) | (0.0156) | (0.0156) | (0.0235) |
|  |  |  |  |  |  |  |  |
| Observations | 81,754 | 77,803 | 74,725 | 72,336 | 30,273 | 26,858 | 18,678 |
| Within R-squared | 0.008 | 0.008 | 0.006 | 0.007 | 0.019 | 0.021 | 0.033 |
| Number of location-time combination | 2,843 | 2,787 | 2,736 | 2,679 | 2,621 | 2,565 | 2,328 |

Standard errors in parentheses

*** p<0.001, ** p<0.01, * p<0.05

|  | (1) | (2) | (3) | (4) | (5) | (6) | (7) |
| --- | --- | --- | --- | --- | --- | --- | --- |
| Outcome:  Log(normalized absolute error) | Week 8 | Week 9 | Week 10 | Week 11 | Week 12 | Week 13 | Week 14 |
|  |  |  |  |  |  |  |  |
| Non-mechanistic | 0.108*** | 0.157*** | 0.145*** | 0.103*** | 0.0897** | 0.0625 | 0.0771* |
|  | (0.0221) | (0.0300) | (0.0305) | (0.0304) | (0.0313) | (0.0329) | (0.0354) |
| Mechanistic with state-resetting | -0.141*** | -0.127*** | -0.139*** | -0.127*** | -0.105*** | -0.169*** | -0.218*** |
|  | (0.0208) | (0.0249) | (0.0254) | (0.0254) | (0.0266) | (0.0283) | (0.0305) |
| Academia | 0.157*** | 0.0568 | 0.0862 | -0.0232 | -0.0589 | -0.289 | 0.177** |
|  | (0.0474) | (0.0501) | (0.0493) | (0.0510) | (0.0944) | (0.172) | (0.0576) |
| Model documentation | 0.174*** | -0.0686 | -0.103 | 0.112 | 0.154 | 0.445* |  |
|  | (0.0319) | (0.0575) | (0.0570) | (0.0631) | (0.104) | (0.180) |  |
| Academia*Model documentation | -0.321*** |  |  |  |  |  |  |
|  | (0.0556) |  |  |  |  |  |  |
| Constant | -11.36*** | -11.31*** | -11.22*** | -11.27*** | -11.19*** | -11.18*** | -11.12*** |
|  | (0.0273) | (0.0389) | (0.0396) | (0.0429) | (0.0448) | (0.0515) | (0.0607) |
|  |  |  |  |  |  |  |  |
| Observations | 15,527 | 8,883 | 8,303 | 7,769 | 7,097 | 6,297 | 5,822 |
| Within R-squared | 0.018 | 0.016 | 0.018 | 0.016 | 0.012 | 0.021 | 0.027 |
| Number of location-time combination | 2,271 | 2,192 | 2,128 | 1,948 | 1,894 | 1,825 | 1,721 |

Standard errors in parentheses

*** p<0.001, ** p<0.01, * p<0.05
