# Supplementary material for: Enhancing long-term forecasting: Learning from COVID-19 models
Source: PLoS Comput Biol. 2022 May 19;18(5):e1010100. doi: 10.1371/journal.pcbi.1010100 (PMC9119494; doi:10.1371/journal.pcbi.1010100)
Supplement: S3 Text — The performance of SEIR-b models are compared with CDC hub models based on a variety of alternative metrics. Fig A. Comparison of performance of models based on head-to-head win fraction. Fig B. Comparison of performance of models based on normalized error relative to constant model. Fig C. Comparison of performance of models based on population-normalized absolute error (DOCX) [file pcbi.1010100.s003.docx]

## S3 Text: Comparison of models based on other measures

In the paper we reported the model rankings based on regressions conducted for predictions at every projection horizon between 1 and 20 weeks. Those regressions include fixed effects for every combination of location and projection date, ensuring that idiosyncratic challenges in projecting specific locations and weeks is not driving the differences in prediction errors across different models. After controlling for those fixed-effects the coefficient for each model represents the distinct contribution of that model to prediction errors. In fact, most models do not offer projections for every location, prediction date, or horizon, making such controls important for fair comparisons across models. Nevertheless, more direct comparisons of measures of prediction accuracy could inform more familiar ways to read the prediction data, and therefore present three of those comparisons below, followed by a replication of the ranking graph including the model names which were not part of the graph in the main paper. Whereas the ranking graph includes only models with 50 predictions for a given horizon, for completeness the three graphs below include all models regardless of number of predictions. This may lead to some outliers, e.g. QJHong-Encounter has submitted fewer than 50 in any horizon, and thus does not show up in the rankings graph, but performs very well where it has submitted a prediction as can be seen in the following graphs.

**Head-to-Head Win Fraction**

For each location-week-horizon combination a few models may offer predictions, offering opportunities to see how models compare against each other in head-to-head battles. For example, if 5 different models are predicting deaths for the week of March 14, 2021 as part of a 10-week ahead horizon, those comparisons offer 4 win/lose options for each model in that set. A model that wins 3 of those 4 head-to-head comparisons gets a score of 0.75 from this location-week. For each model such win fractions, when averaged across all such comparisons for the 10-week horizon, would inform the quality of the model’s predictions at 10-week ahead horizon. The following graph reports those average fractions across CDC model set and SEIRb family. Using this measure SEIRb outperforms other models in several longer time horizons while QJHong-Encounter (when it submitted a prediction), YYG-ParamSearch and IHME also perform very well (Fig A).


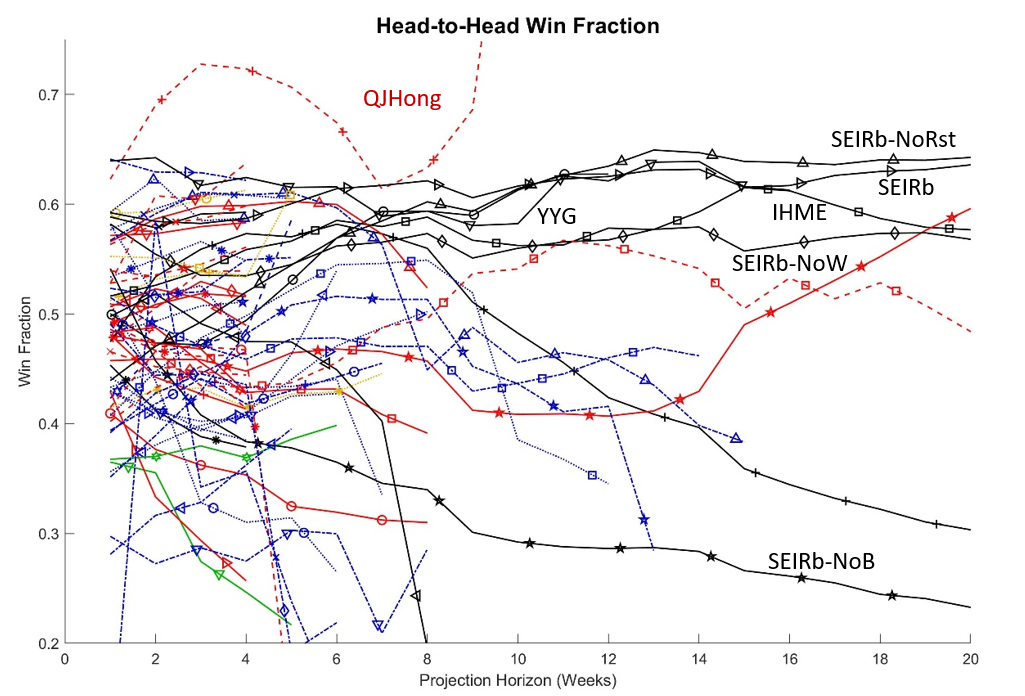


Fig A: Comparison of performance of models based on head-to-head win fraction

**Normalized Error Relative to Constant Model**

The next performance measure compares various models against the naïve (constant) benchmark. As discussed in the paper, the constant benchmark is not that naïve after-all: it is the straightforward prediction that accounts for endogenous behavioral feedback keeping the reproduction number around 1; it also beats many models both in the short and long-term horizons. Specifically, for each model we go through the following calculations: for each prediction (for a given location, week, and horizon) the per capita error for the constant model is deducted from the model’s per capita error to offer a comparative normalized error; then the median across all those comparative errors for each projection horizon is mapped (median is used given the fat-tailed distribution of these errors). Using this measure IHME and SEIRb are the top performers in longer time horizons (Fig B) while Caltech-CS156, MSRA-DeepST, and QJHong-Encounter (when submitting a prediction), offer the best short-term predictions.


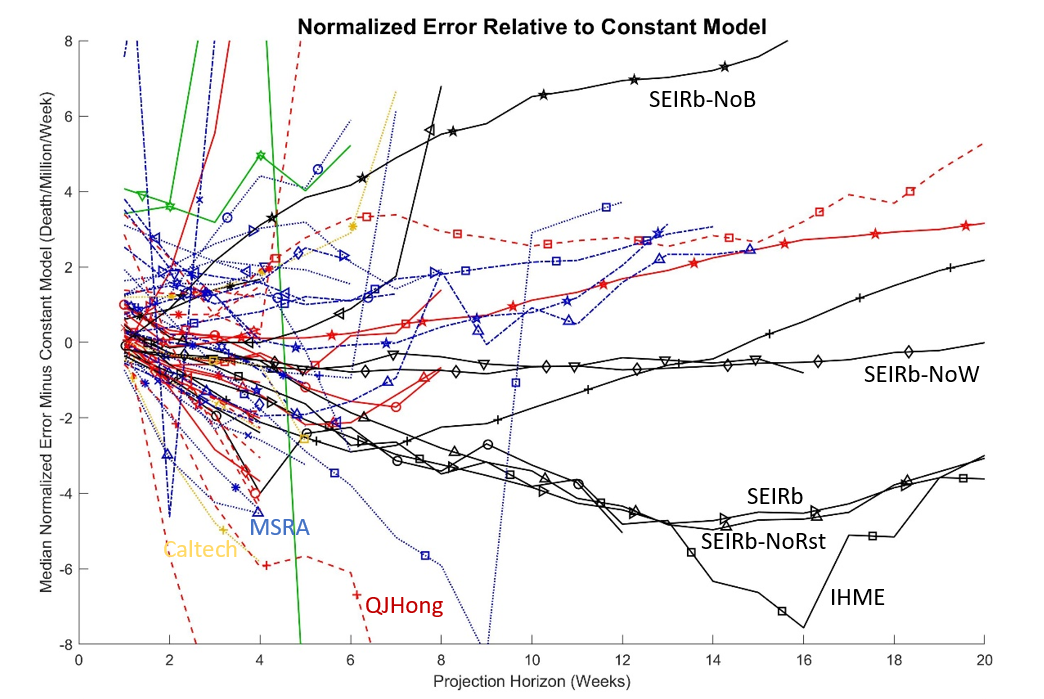


Fig B: Comparison of performance of models based on normalized error relative to constant model

**Absolute Prediction Error (Population Normalized)**

The next detailed graph reports the absolute prediction error (normalized by location populations, in Death/Million/Week). For each model and each horizon, we report the median error across all locations and projection dates for which that model has submitted a prediction. It is noteworthy that this metric leads to somewhat different rankings compared to other measures: because each model has submitted predictions for only a subset of locations, projection dates, and horizons some may be competing on harder forecast tasks than others. This problem was partially addressed in the first two graphs by comparing models against each other (in Win fraction measure) or comparing against a constant model (in the second graph above). It was also more explicitly addressed by including fixed effects (comparing against mean) for each location-projection date-horizon in the primary regressions (used in the main ranking graph).


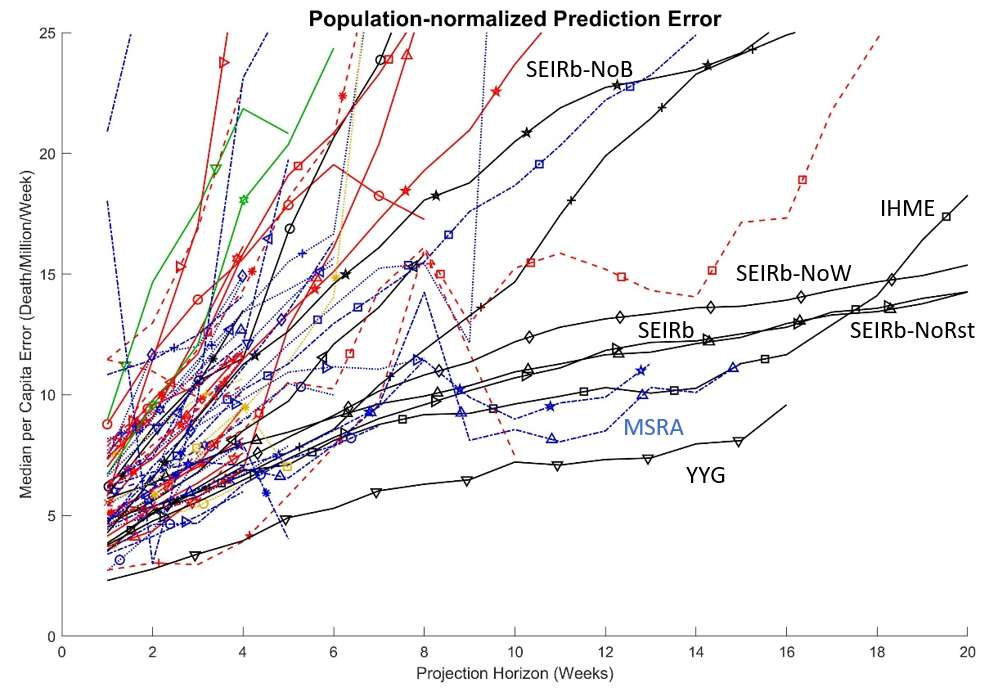


Fig C: Comparison of performance of models based on population-normalized absolute error
