# Supplementary material for: Enhancing long-term forecasting: Learning from COVID-19 models
Source: PLoS Comput Biol. 2022 May 19;18(5):e1010100. doi: 10.1371/journal.pcbi.1010100 (PMC9119494; doi:10.1371/journal.pcbi.1010100)
Supplement: S4 Text — The details of simulation model formulation, calibration and improvement opportunities are explained. Table A. List of parameters estimated in model calibration and their search (feasible) ranges. (DOCX) [file pcbi.1010100.s004.docx]

## S4 Text: Model Documentation

## Model formulation

For the purpose of parsimony, we develop a very simple model. Consistent with conventional SEIR models, the population ($N$) is represented in four stocks of Susceptible ($S$), Exposed ($E$), Infectious ($I$), and Removed ($R$) (eq. 1-4).

$\frac{dS}{dt}=-\frac{\beta SI}{N}$

$\frac{dE}{dt}=\frac{\beta SI}{N}-\frac{E}{\tau_{1}}$

$\frac{dI}{dt}=\frac{E}{\tau_{1}}-\frac{I}{\tau_{2}}$

$\frac{dR}{dt}=\frac{I}{\tau_{2}}$

(1-4)

where $\beta$ is transmission intensity, $\tau_{1}$ is exposure period, and $\tau_{2}$ is infection period from symptom onset to recovery or death. In this simple representation, daily death of $f$ can be represented as a fraction of removal rate, where the fraction, $i$, is referred as infection fatality rate (eq. 5)

$f=i\frac{I}{\tau_{2}}$ (5)

The transmission intensity of $\beta$ which determines the speed of the spread of the disease, and the reproductive number, should change overtime, and in fact that is the main difference between our SEIRb model and others. Specifically, we expect $\beta$ to decline and people practice more NPIs as perceived risk of death ($f'$) increases, i.e., $\frac{d\beta}{df^{'}}<0$. Consistent with the literature that finds change in weather influences transmission [30], we include weather impact of $w$ in the formulation of transmission intensity. Equation 6 shows how we represented this relation in one of its simplest formats:

$\beta=\beta_{0}w\frac{1}{\left( 1+\alpha f' \right)^{\gamma}}$ (6)

For the weather impact of $w$, projections from a previous study [30] is used in our comparisons. Specifically, we start with the “Covid-19 Risk factor due to Weather” (CRW) that was publicly released in May 2020, and use a transformation of that factor ($w=CRW^{2.64}$) based on other modeling work [18] that had found the CRW factor to be conservative in reflecting the impact of weather on transmission.

Equation 6 closes a balancing feedback loop from daily death rate to future transmission intensity and consequently future exposure, onset, and death. In this relation, $f'$ is simply modeled as a lagged variable of $f$, daily deaths, assuming public risk perception is a lagged function of confirmed death cases. Perception adjustment for increasing and decreasing death may be different, thus we include two lag times for upward ($\tau_{U}$) and downward ($\tau_{D}$) adjustment of $f'$, both estimated from model calibration:

$\frac{df^{'}}{dt}=\frac{f-f^{'}}{\tau_{R}}$

$\tau_{R}=\left\{ \begin{matrix} \tau_{U} if f>f' \\ \tau_{D} if f\leq f^{'} \end{matrix} \right.$ (7)

## Model Calibration

Model calibration is done separately and independently for each location (53 states and territories of USA with population over 200,000) and estimation/projection date (T_f_: the end of each Saturday starting on May 2, 2020 and ending on March 13 2021). Estimation is pursued maximizing the likelihood of observed Cases and Deaths for each location starting from an initial time for data inclusion (T_0_: when the official number of cases/deaths respectively exceeds 1e-6/1e-8 per day as a fraction of location’s population, or the beginning of May 2020, whichever comes first) until the estimation/projection date (T_f_). We use a Negative Binomial likelihood function for both cases and deaths (x_vt_ and y_vt_($\theta$): where x is data, y($\theta$) is model predictions for data given the unknown parameter vector $\theta$; t is the day and $v\in[i,d]$ denotes the cases/deaths; we smooth out weekly cycles by using 7-day moving averages for death data):

$LL\left( \theta,\lambda_{v} \right)=\sum_{v} \sum_{t=T_{0}}^{t=T_{f}} -\frac{\ln\left( 1+\lambda_{v}x_{tv} \right)}{\lambda_{v}}+ln\Gamma\left( x_{tv}+\frac{1}{\lambda_{v}} \right)-ln\Gamma\left( \frac{1}{\lambda_{v}} \right)-\left( x_{tv}+\frac{1}{\lambda_{v}} \right)\ln\left( 1+\lambda_{v}y_{tv}\left( \theta\right) \right)+x_{tv}(\ln\left( y_{tv}\left( \theta\right) \right)+\ln\left( \lambda_{v} \right))$ (8)

In this function $\Gamma$(z) represents the natural logarithm of the generalized factorial function for z-1 ($ln\Gamma\left( z+1 \right)=ln(z!)$ for integer z). Predicted deaths ($y_{td}\left( \theta\right)$) come directly from the SEIR model described above.

Two additional features inform the estimation process (but not projections, which come purely from the SEIRb model described above). First, before the projection date (T_f_) the perceived risk, $f^{'}$, uses the actual data ($x_{td}$) rather than simulated values for deaths. That is we use the following equation instead of equation 7:

$\frac{df^{'}}{dt}=\frac{x_{td}-f^{'}}{\tau_{R}}$ (9)

Second, we use the following equations to predict cases ($y_{ti}\left( \theta\right)$):

$y_{ti}\left( \theta\right)=\frac{\beta SI_{D}}{N}$ (10)

$\frac{dI_{D}}{dt}=x_{ti}-\frac{I_{D}}{\tau_{2}}$ (11)

Essentially, the data for measured infections flows into a stock (I_D_) that parallels the model-simulated infection rate (that is, it flows out with the same time constant of $\tau_{2}$), and this stock of “measured” infectious population is used to predict expected “measured” infections based on model generated transmission intensity and susceptible fraction. This approach enables using measured case data to inform the parameters going into transmission intensity (most notably the response function parameters) without worrying about ascertainment rates which likely are far below 100% and vary across locations.

The vector of estimated model parameters ($\theta$) and the ranges we use for each in the calibration are listed in Table A.

Table A. List of parameters estimated in model calibration and their search (feasible) ranges.

| Parameter | Range | Units | Explanation |
| --- | --- | --- | --- |
| $\beta_{0}$ | [0.1-4] | 1/Day | Basic Transmission Intensity |
| $\alpha$ | [0.01,100] | Day/Person | Sensitivity to death |
| $\gamma$ | [0,5] |  | Death risk diminishing impact |
| $\tau_{U}$ | [1,100] | Day | Time to adjust risk perception upwards |
| $\tau_{D}$ | [10,400] | Day | Time to adjust risk perception downwards |
| T_0_ | Oct 15, 2019-Mar 3, 2020 | Day | Patient zero arrival time |
| I | [0.003-0.01] |  | Infection fatality rate |

We also estimate two parameters regulating the shape of the negative binomial distribution ($\lambda_{v}$), leading to a total of 9 estimated parameters for each location and estimation/projection date for the SEIRb model. Other variants include the same or fewer parameters (SEIRb-NoB: 5; SEIRb-NoW: 9; SEIRb-NoRst: 9) but otherwise follow the same exact calibration process.

Maximization of the likelihood function in equation 8 is pursued using Powell Direction Set method built into Vensim simulation software. For each location we conduct one initial calibration for the last estimation date (March 13, 2021) with 15 different random start points for unknown parameters. For all other estimation dates we use 5 different start points but also include the parameter setting found in the next estimation date. This process enhances our confidence in finding good optimization solutions while keeping the computational costs to a minimum. Overall all the 2436 (=53*46) estimations for SEIRb model could be completed in about 4 hours on a regular desktop when compiled and parallelized over 10 cores.

## State resetting

The basic idea of state resetting is to ensure projections start from the right level given the most recent data on cases and deaths. Various data fusion, smoothing, and filtering methods exist to leverage current data to offer good, even optimal, estimates for state variables in a model. Those methods can enhance both model estimation and projections, however, they are computationally expensive and their elaborate setup may mask the basic benefits achievable from more simple state resetting schemes. We therefore opt for using a simpler approach in which only once, at T_f_, we reset the two relevant state variables of E and I to their likely values, E* and I*, given recent deaths and cases. Specifically, we use the following equations to calculate E* and I*:

$E^{*}=\frac{x_{d}^{*}\left( 1+s_{E} \right)}{i}\tau_{1}$

$I^{*}=\frac{x_{d}^{*}\left( 1+s_{I} \right)}{i}\tau_{2}$

$\frac{dx_{v}^{*}}{dt}=\frac{x_{tv}-x_{v}^{*}}{\tau_{a}}$ ; $\tau_{a}=7$ days

$s_{E}=w_{dE}\sigma_{d}\left( \frac{\tau_{1}}{2}+\tau_{2} \right)+(1-w_{dE})\sigma_{i}\left( \frac{\tau_{1}}{2}+\tau_{a} \right)$

$s_{I}=w_{dI}\sigma_{d}\left( \frac{\tau_{2}}{2}+\tau_{a} \right)+(1-w_{dI})\sigma_{i}\left( \tau_{1}+\frac{\tau_{2}}{2}+\tau_{a} \right)$

$w_{dI}=\frac{\frac{2}{\tau_{2}}}{\frac{1}{\tau_{1}+\frac{\tau_{2}}{2}}+\frac{2}{\tau_{2}}}$

$w_{dE}=\frac{\frac{1}{\tau_{1}+\frac{\tau_{2}}{2}}}{\frac{1}{\tau_{1}+\frac{\tau_{2}}{2}}+\frac{2}{\tau_{2}}}$

$\sigma_{v}=\frac{x_{tv}-x_{v}^{*}}{\tau_{a}\left| x_{v}^{*} \right|}$

(12-19)

The basic idea behind these equations is to calculate expected E and I state variables based on (recent) death rate ($x_{d}^{*}$) and adjust that approximation based on the expected slope of E and I (s_E_ and s_I_) calculated using the observed slopes of cases ($\sigma_{i}$) and deaths ($\sigma_{d}$).

## Important simplifications and improvement opportunities

The SEIRb model is very simple. It is built only to test the usefulness of three features we find correlate with the predictive quality of various models, and by design, to exclude various other features which could further enhance a predictive model. Here we provide a partial list of those missing features, focusing on mechanistic models (elaborating on alternative curve-fitting models goes beyond the scope of this paper). Since we have not tested the features below we cannot comment on their relative value in terms of enhancing predictive power, but we suspect several from this list could improve upon SEIRb’s performance. Indeed, the model “IHME-CurveFit” outperform SEIRb over longer time horizons, and benefit from incorporating a few of these features. However, several other models do benefit from a subset of these features and yet do not show notable improvements over SEIRb, thus we do not imply that incorporating all these features would tend to enhance a model’s predictive power.

Model Structure

- Capturing operational mechanisms of relevance
  - Loss of immunity among those recovered, reinfections, and potential reduction in severity of disease in future infections
  - Hospitalization, treatment, and critical care capacity
  - Testing, changes in testing capacity, and its impact on ascertainment and risk response
  - Changes in demand for testing based on recent cases and deaths
  - Prioritization of testing and treatment capacity based on symptoms and other factors
  - Incorporating travel networks between different locations and importation of cases from abroad
- Modeling at more granular levels
  - Modeling at county (vs. state) level
  - Disaggregating based on age groups and high vs. low risk groups
  - Disaggregating based on severity of disease, including asymptomatic transmission
- Capturing additional feedback mechanisms
  - Changes (reductions) in Infection Fatality Rate with accumulation of deaths due to changes in behavior among higher risk groups (e.g. elderly), improved treatment, and depletion of most at-risk populations (e.g. nursing homes).
  - Changes in behavioral response due to adherence fatigue
  - Emergence of new variants and endogenous changes in transmissibility of the SARS-CoV-2 virus

Data Sources

- Including data for testing
- Including data for hospitalization and ICU visit
- Including data for mobility changes in each location
- Incorporating data for mobility across locations
- Incorporating data for government policies and mandates, and their removal over time

Model Estimation

- Representing delays in reporting of cases and deaths
- Estimating various assumed model parameters (e.g. $\tau_{1},\tau_{2},\tau_{a}$)
- Estimating the impact of weather factors on transmission directly
- Jointly estimating model parameters across states, using hierarchical Bayesian methods
- Using more sophisticated likelihood functions to account for interdependency over time and across locations in the observed data
- Using more sophisticated optimization algorithms and more computational power to decrease the chances of converging to local peaks in the parameter space

Fine tuning for prediction

- Testing alternative model structures to pick the one that offers better predictions
- Testing alternative assumed parameters to pick the set offering better predictions
- Adopting different model structures for different locations to enhance prediction
- Adopting ensembles of models to increase predictive robustness

State resetting

- Using particle filters, extended or unscented Kalman filters, or other filtering methods for state resetting
- Also resetting other state variables (e.g. perceived risk) based on recent cases and deaths
