# Supplementary material for: Enhancing long-term forecasting: Learning from COVID-19 models
Source: PLoS Comput Biol. 2022 May 19;18(5):e1010100. doi: 10.1371/journal.pcbi.1010100 (PMC9119494; doi:10.1371/journal.pcbi.1010100)
Supplement: S5 Text — Models’ performance using alternative metrics as well as by different periods are explored. Fig A. Death projection performance of the CDC model set over different time horizons compared to a constant model based on Interval Score. Fig B. Death projection performance of the CDC model set over different time horizons compared to a constant model before turning points (a) and after turning points (b). Fig C. Forecast quality ranks for CDC model set and SEIRb (a) pre- and (b) post- turning points. (DOCX) [file pcbi.1010100.s005.docx]

## S5 Text: Additional Analyses on Model Performance

In this section we further assessed the models’ performance using alternative metrics as well as by different periods. First we adopted Interval Score (IS) based on the 95% prediction interval as the outcome [22], which summarizes each 95% prediction interval as a single number and is penalized by not containing true death as well as wide intervals. We do not use this metric in the main analysis because determinants of confidence interval accuracy are not the topic of our study. We replicated our main analysis for study 1 (see details in S2 Text) comparing IS (normalized by state population) for each model in the CDC repository. As constant model only contains point estimates, we constructed the width of its prediction interval in each location-horizon-projection date combination to be the median of the prediction interval width for all CDC models in the same location-horizon-projection date combination. This choice does not impact how the performance of CDC models in different categories compare to each other. Results were summarized in Fig A, which were largely consistent with the one in the main paper, but showed a larger advantage in model performance for compartmental models with state-resetting (compared to other model categories).

Fig A: Death projection performance of the CDC model set over different time horizons compared to a constant model based on Interval Score.

To provide insights on whether timing of the prediction impact each model’s performance and if certain models are better at predicting turning points (e.g., emergence of a new peak), we explored each model’s performance for two subsets of forecasts, based on whether they were made pre- or post- turning points. Specifically, we divided the death time series for each location into different segments marked by turning-points in the (smoothed) death rates: each segment starts from one turning-point (maximum/minimum) and ends at the next. We then divided all predictions into two groups: those where the prediction date (the date at which prediction is done) and target date (the date for which a model is predicting the number of deaths) are within the same segment (pre-turning) or in different segments (post-turning). We replicated our analyses in Fig 1B and Fig 3C in the main text for each group. This approach allows us to quantitatively assess the types of models that are better in predicting death before/after reaching the turning points (extremums).

Fig B compared the performance of all models in CDC repository and the results showed that: (1) as expected, all models performed worse when there were turning points in upcoming death trends, but compartmental models with state-resetting still performed best overall; (2) as before compartmental models without state-resetting performed worse than non-mechanistic models in the short term, and their advantage grew with forecast horizon; however, this trend was more salient for predictions post-turning points. We concluded that there might be some evidence about additional benefits of compartmental models in forecasts that should foresee upcoming turning points but this evidence is at best indicative.

Fig B. Death projection performance of the CDC model set over different time horizons compared to a constant model before turning points (a) and after turning points (b) (week 1 was excluded from (b) for consistent scales on Y-axis across figures).

We also replicated the analysis in Fig 3C in the main text to evaluate how performance of SEIR-b and related models compared with other models in CDC repository based on the same definitions of segments and pre- vs. post- turning point forecasts. The results in Fig C showed that the top performing models (IHME and SEIRb) remain at the top for the longer time horizons in both pre- and post-turning points. Thus we found no strong support for the hypothesis that behavioral models are distinctly better post-turning points.


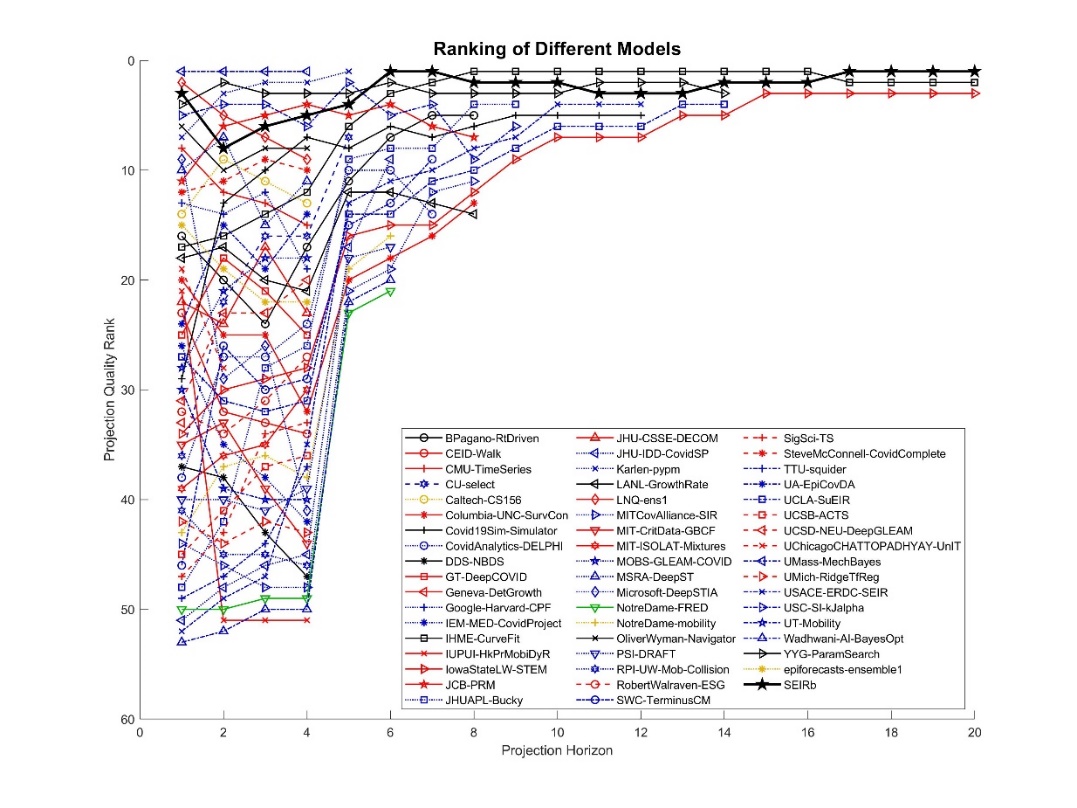


**(pre- turning points)**


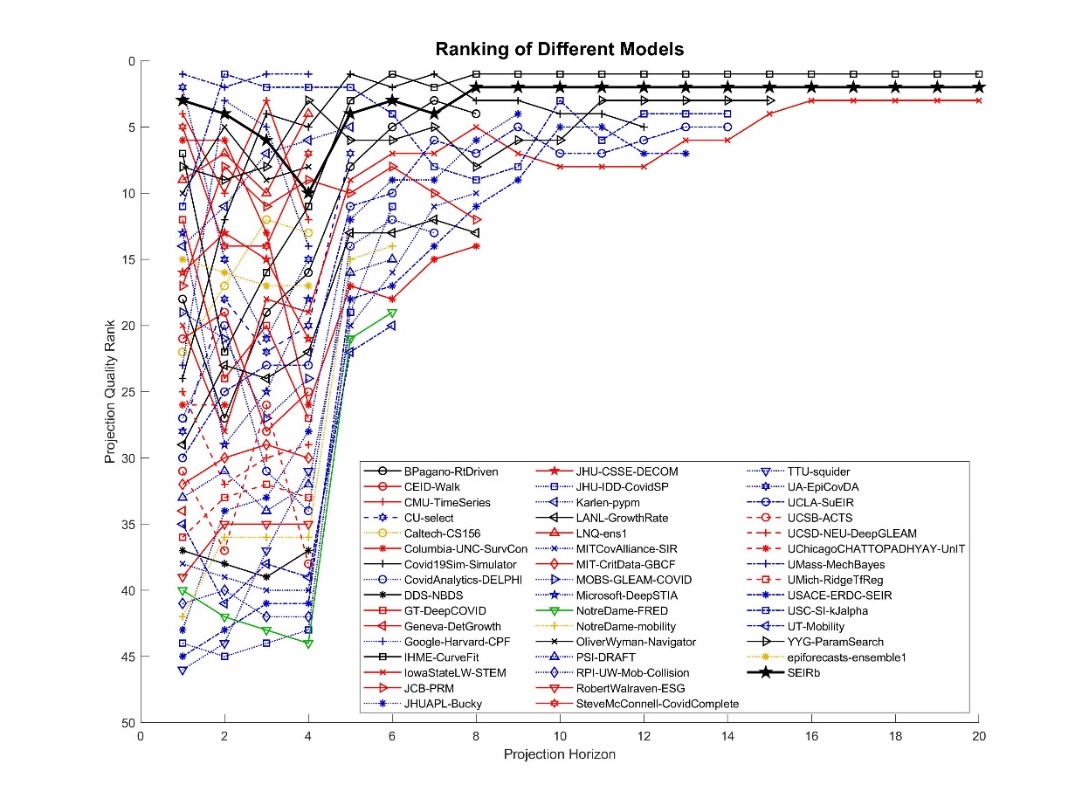


**(post- turning points)**

Fig C. Forecast quality ranks for CDC model set and SEIRb based on regressing Ln(Per capita projection error) against models, controlling for location-horizon-week combinations (a) pre- and (b) post- turning points. Color codes: compartmental models without state-resetting (blue); with state-resetting (black); non-mechanistic (red); agent-based (green); and ensemble (yellow).
